# Investigation of the Impact of Graphene Nanoplatelets (GnP) on the Bond Stress of High-Performance Concrete Using Pullout Testing

**DOI:** 10.3390/ma14227054

**Published:** 2021-11-20

**Authors:** Fouad Ismail Ismail, Yassir M. Abbas, Nasir Shafiq, Galal Fares, Montasir Osman, Lotfi A. Hussain, Mohammad Iqbal Khan

**Affiliations:** 1 Department of Civil and Environmental Engineering, Universiti Teknologi PETRONAS, Seri Iskandar 32610, Perak, Malaysia; fouad_20001008@utp.edu.my (F.I.I.); nasirshafiq@utp.edu.my (N.S.); montasir.ahmedali@utp.edu.my (M.O.); 2 Department of Civil Engineering, Faculty of Engineering, Al-Azhar University, Cairo 11884, Egypt; 3 Department of Civil Engineering, King Saud University, Riyadh 800-11421, Saudi Arabia; yabbas@ksu.edu.sa (Y.M.A.); galfares@ksu.edu.sa (G.F.); lotfe_abo7oseen@hotmail.com (L.A.H.)

**Keywords:** graphene nano platelets, high-performance concrete, bond stress, bond stress-slip behavior, pullout test, failure mode

## Abstract

Efficient load transmission between concrete and steel reinforcement by bonding action is a key factor in the process of the design procedure of bar-reinforced concrete structures. To enhance the bond strength of steel/concrete composites, the impact of graphene nanoplatelets (GnP) on the bond stress and bond stress–slip response of deformed reinforcement bars, embedded in high-performance concrete (HPC), was investigated using bar pullout tests. In the current study, 36 samples were produced and examined. The main variables were the percentages of GnP, the steel reinforcement bar diameter, and embedded length. Bond behavior, failure mode, and bond stress-slip response were studied. Based on the experimental findings, the inclusion of GnP had a significant favorable influence on the bar-matrix interactions due to the bridging action of GnP as a nano reinforcement. For 0.02 wt.% of GnP, the bond strength was enhanced by more than 41.28% and 53.90% for steel bar diameters of 10 and 16 mm, respectively. The HPC-GnP mixture displayed a reduction in the initial slippage in comparison to the control sample. The test findings were compared to the prediction models created by other researchers and the ACI 408R-12 code.

## 1. Introduction

Concrete, the most widely used building material, has long been able to meet the growing needs of mankind. Concrete buildings have long been made up of many types of concrete, such as normal concrete, high-strength concrete (HSC), and high-performance concrete (HPC). For its improved mechanical characteristics and durability, HPC is increasingly used in high-rise buildings, bridges, and offshore constructions. High-strength concrete is described as concrete with a low water/binder ratio and an adapted aggregate-to-binder ratio to regulate its dimensional stability (i.e., drying shrinkage) and adequately water-cured (to control autogenous shrinkage) [1]. The investigation of reinforced HPC with diverse materials has received much attention. A substantial amount of effort has gone into researching the different material characteristics of HPC. In this sense, the growing technique for utilizing industrial products to develop reinforced HPC has motivated the interest of researchers in recent years.

**Figure 1:**
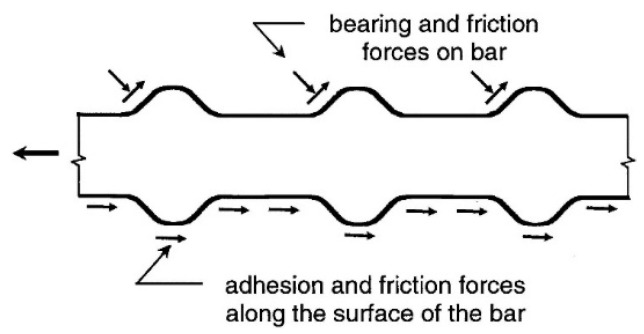
Bond force transfer mechanisms [2].

For optimal design in reinforced concrete structures, effective and dependable force transmission between reinforcement and concrete is essential [2,3]. Bonded concrete is a key structural characteristic that ensures strain compatibility and composite action to transmit stresses between concrete and the strengthening steel [2]. The insufficient connection might result in a considerable loss of load capacity and structural rigidity [4]. For a deformed bar, chemical adhesion forces are transmitted from the reinforcing steel to the surrounding concrete, this bond causes frictional forces between the steel bar and the adjacent concrete [2]. The mechanical anchorage of the steel bar causes bearing stresses on the ribs against the concrete surface, as indicated in Figure 1 [5].

Previous studies have shown that bond strength is regulated by several parameters [2]. For example, the compressive strength of the concrete, tensile strength, the cover thickness of concrete around the bar, embedded length, reinforcement in the transverse direction that confines concrete, and the bar shape [6,7,8,9,10,11]. One approach to analyzing the bond between concrete and steel is to study the evolution of the bond stress-slip typically achieved using conventional bar pullout tests [12]. Several researchers examined the bond strength of HSC and HPC. For instance, Orangun et al. [3], ACI committee 408R [2], Hadi [13], and Chapman and Shah [14] proposed equations based on the compressive strength, side cover of the bar, bar diameter, and embedded length. Additionally, Esfahani and Rangan [6] proposed an equation that considers the side cover of concrete, embedded length, and the concrete tensile strength.

On the contrary, current research efforts focus on finding new methods to improve concrete performance by nanoengineering the physicomechanical and chemical characteristics of cement, which is the major binding ingredient in the mix [15]. Nanomaterials have been successfully incorporated into various products, as a result of advances in nanotechnology, including nano-CaCO_3_ [16], nano-SiO_2_ [17], and nano-TiO_2_ [18], as reinforcing materials in cement to prevent crack propagation at the nanoscale. Nano-cracks with their large aspect ratio have proved to be efficiently arrested by carbon nanotubes (CNT) and carbon nanofibers (CNFs) [19,20]. Konsta et al. [21] reported that due to the ability of nano-reinforcements to manage nanosized cracks (at the initiation stage) before they grow into micro-sized cracks, nano-reinforcements in cementitious materials are more effective than traditional steel bar/fiber reinforcements (at mesoscale).

A new possibility for nanosized cementitious additives has emerged with the recent discovery of graphene [22,23], which may be employed in cementitious materials. Graphene nanoplatelets (GnP) and their oxides, particularly graphene oxide nanoplatelets (GONPs), are two kinds of graphene-based nanomaterials that are both low-cost nanoparticles [24] made up of graphene stacks [25,26]. Graphene nanoplatelets have a 2D sheet shape with a nano-scale thickness (less than 10 nm). In addition to their inherited benefits from graphene, GnP also promises nano-sized additions and perfect reinforcement for structural materials.

A handful of recent research showed that the inclusion of graphene nanoplatelets in cementitious composites exhibited excellent mechanical properties. The review study of Rehman et al. [27] demonstrated that graphene significantly improved the mechanical properties of cement-based composites. Additionally, the study of Peyvandi et al. [28] resulted that the flexural strength of cement matrix may be increased by 27% to 73% by incorporating different types of GnPs and their oxides into the cement matrix at a rate of 0.13 wt.%. Moreover, Chuah et al. [29] revealed that low concentrations of GONPs could enhance cement paste’s compressive strength by 46.2%. Furthermore, adding GONPs to cement at a level of 0.05 wt.% has resulted in a 15–33% improvement in compressive strength; in addition, the flexural strength has been increased by 41–59% [30]. Additionally, Gong et al. [31] reported that with GONPs within GONP/cement composite at 0.03 wt.%, compressive strength and tensile strength might be enhanced by more than 40%; also, the cement paste’s total porosity was reduced. Furthermore, Mokhtar et al. [32] reported that with 0.02 wt.% and 0.03 wt.% of GONPs, the compressive and indirect tensile strengths were improved by 13% and 41%, respectively. Moreover, Rehman et al. [33] indicated that GnP of 0.03% was able to enhance the load capacity and failure strain by 30 and 73%, respectively. Likewise, Meng et al. [34] examined the impact of graphite nanoplatelets (GNPs) and carbon nanofibers (CNFs) on the mechanical characteristics of ultrahigh-performance concrete (UHPC). It was reported that flexural strength and toughness were enhanced by 59% and 276%, respectively, with the inclusion of 0.30% GNPs. Moreover, the tensile strength and energy absorption capacity improved by 40% and 187%, respectively, as the amount of GNPs was raised from 0 to 0.30% [34]. Additionally, Chen et al. [35] indicated that Graphene Oxide (GO) can improve the compressive strength, flexural strength, and elasticity modulus of concrete by 4.04–12.65%, 3.8–7.38%, and 3.92–10.97%, respectively. Furthermore, concrete’s compressive strength may be significantly improved by using GO nanosheets [35]. Likewise, Rehman et al. [36] revealed that the addition of GO nanosheets by 0.03% can increase the compressive strength of cement-based composites by 27%.

In accordance with the reported study by Konsta et al. [21], which reported the ability of nano-reinforcements to manage nanosized cracks. The behavior of GnP can be similar to CNT; therefore, Qasem et al. [37] examined the effect of CNT on the bond behavior between UHPC and steel bars, this research showed that 0.02 wt.% CNT enhanced the maximum bond stress of steel rebars with diameters of 12 mm and 16 mm by 34.7 and 48.5%, respectively.

Reviewing the studies on the impact of graphene nanoplatelets on HPC and bond strength, no appreciable investigation has been conducted. Therefore, in this study, experimental tests and mathematical verification according to the available models were performed. The parameters affecting the bond strength (i.e., bar diameter, embedded length of the bar, and the effect of different dosage percentages of GnP) were examined. In order to accomplish this goal, the HPC compressive strength was first obtained at a curing age of 28 days. Then, 36-cylinder samples were tested for bond-slipping behavior between HPC-GnP and rebar. Finally, the pullout test results were analyzed, and the reliability of available models was studied.

## 2. Experimental Program

In the current investigation, the experimental program was conducted to investigate the bond stress-slip behavior and pullout strength of reinforcement bars embedded in the HPC with GnP. The major variables of this study were: (i) different percentages of GnP by weight of cement (0.00, 0.02, 0.05, 0.10, 0.30, and 0.50%), (ii) the steel reinforcement bar diameter ($d_{b}$ of 10 mm, 12 mm, 16 mm), and (iii) the bar embedded lengths inside the cylinder $(L_{d}$), which were ($9\;d_{b})$ and ($12\;d_{b})$. Details of concrete designations and variables are shown in Table 1.

### 2.1. Materials

In this investigation, Type II Ordinary Portland cement was used to develop the HPC. The cement was graded (CEM II/B-L 32.5N) and conformed to the requirements of ASTM C150/C150M [38]. Silica fume (supplied by Elkem Micro Silica, Oslo, Norway) was used as a replacement for 7% of the binder material. The physicochemical properties of the cement and silica fume were obtained using X-ray fluorescence (XRF) analysis, as given in Table 2 and Table 3. The fine aggregate passed through a 4.75-mm sieve with a fineness modulus of 2.86 and a specific gravity of 2.65. Crushed granite with a size grading of 5 mm to 10 mm [39] was used with a specific gravity of 2.55. A polycarboxylate ether-based superplasticizer was used to obtain the required workability. Sika Viscocrete-2088 supplied by Sika (Baar, Switzerland) was used in the HPC mixes. Tensile tests were performed on the reinforcement bars according to ASTM A370-20 [40]. The yield strength was 520 MPa, while the failure strength was 620 MPa.

**Figure 2:**
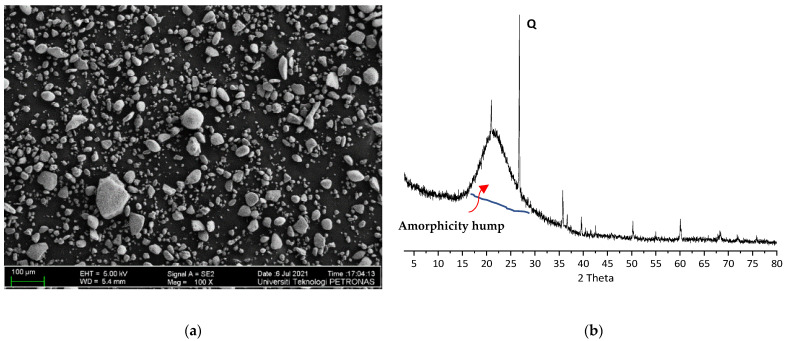
(**a**) FESEM of Silica Fume; (**b**) XRD of Silica Fume (Note. Q: Quartz).

The size of silica fume particles was determined using Field Emission Scanning Electron Microscopy (FESEM) as shown in Figure 2a. The result of X-ray diffraction (XRD) analysis is shown in Figure 2b. XRD analysis of silica fume shows the presence of a hump at the 2-theta range of 15 to 27 degrees. This hump implies that most of the silica fume particles are amorphous SiO_2_.

**Figure 3:**
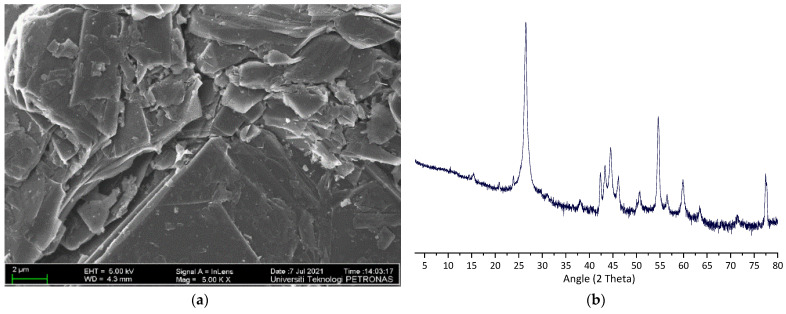

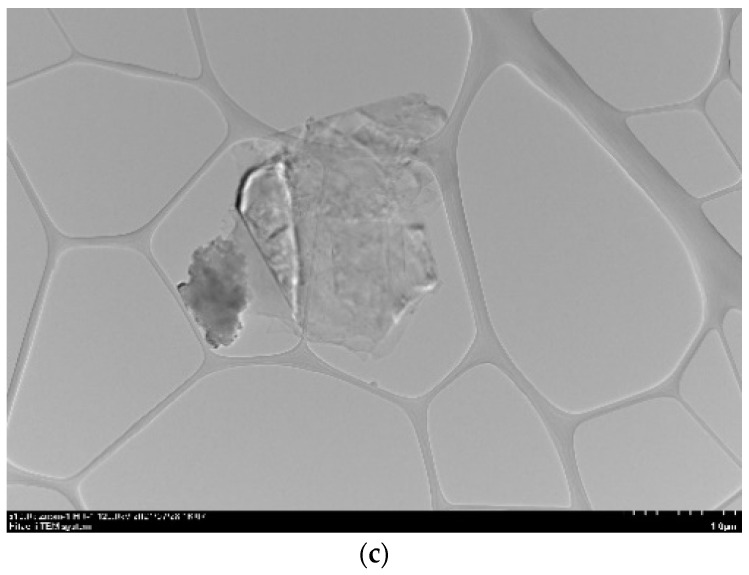
(**a**) FESEM for GnP; (**b**) XRD for GnP; (**c**) TEM for GnP.

Commercially available GnP was supplied by XG Sciences, Inc. (Lansing, MI, USA). Table 4 shows the physical properties of (xGnP-C300). Figure 3 represents the XRD, FESEM, and TEM patterns of the GnP. The XRD pattern of GnP powder showed that the characteristic peaks were at 26.50°, 42.31°, and 54.60° with high intensity. Additionally, the FESEM and TEM showed the particle size of the employed GnP.

### 2.2. HPC Mix Proportions

**Figure 4:**
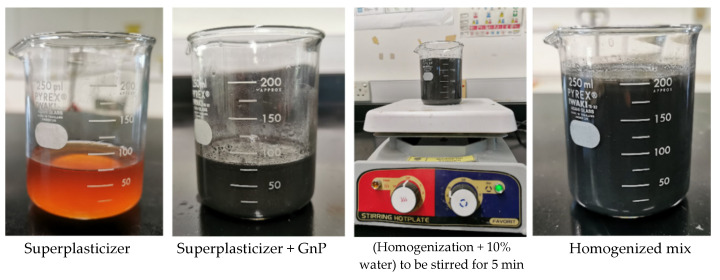
Descriptive method of the dispersion process of GnP.

The ACI guidelines 211.1 revised by Aïtcin [39] were considered for the design of the HPC of M60 Grade. The details of mix proportions are given in Table 5. Figure 4 shows the dispersion process of GnP. To achieve proper GnP dispersion, the required GnP amount for each mix was added to the weighted required superplasticizer plus 10% of the required water and mixed using an electric stirrer for 5 min [41,42,43]. The weighted aggregate, cement, and silica fume were placed in the mixer and combined for two minutes to ensure the correct mixing of solid particles. Therefore, the remaining water was added to the mix and stirred for another two minutes. The superplasticizer and the suspended GnP were then added to the solution and stirred for an additional two minutes to achieve the optimal GnP distribution. The mixing was done following ASTM C192/192M [44].

### 2.3. Pullout Specime’s Details

**Figure 5:**
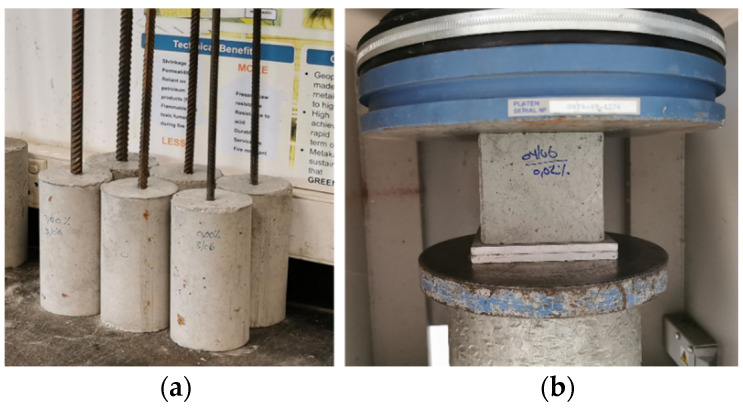
Experimental HPC-GnP specimens prepared for tests: (**a**) the pullout; (**b**) compressive strength.

**Figure 6:**
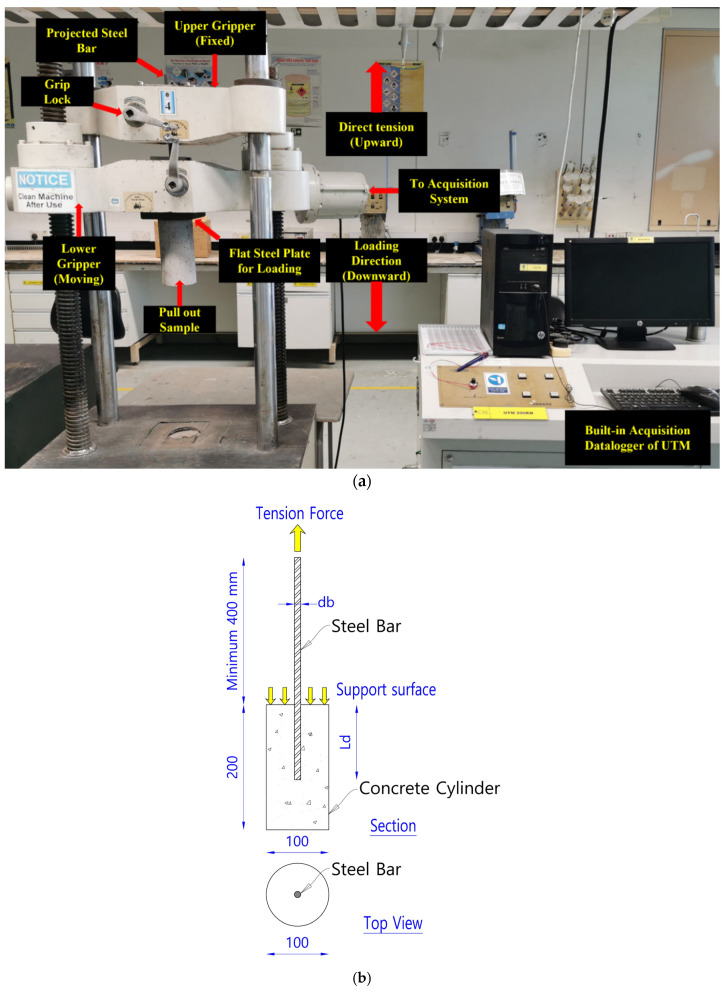
Test setup and specimen geometry: (**a**) the setup for the direct tension pullout test; (**b**) test direction, and specimen geometry.

Tests were performed on a sample size of 36 to determine the bond stress of the HPC-GnP combination. Figure 5 shows the cylindrical and cubic samples used in this study. The specimens consisted of a single reinforcing bar embedded vertically in the middle of each of the concrete cylinders. As indicated in Figure 6, the bar was projected from the top of the cylinder by about 400 mm upwards to provide a sufficient length to grab the specimen in the testing machine. Steel cylinder molds of size 100 mm (dia.) and 200 mm (ht.) were used for all test specimens. The molds were cast in three layers and vibrated adequately along with the embedded steel bar on the vibrating machine. The top level of the specimen was leveled to obtain a smooth surface. The molds were removed after 24 h, and the specimens were kept at a lab temperature of 25° for 28 days. Additionally, cubes of size 100 mm samples were cast to obtain the compressive strength of each mix of concrete according to ASTM C39/C39M-21 [45]. An average of three specimens of cube samples was considered for the compressive strength, as listed in Table 6.

### 2.4. Pullout Testing

The pullout test was performed using a Universal Testing Machine (UTM) with a capacity of 200 kN. The monotonic loading speed employed in this study was 0.367 kN/s. The loading and total displacement parameters were acquired using a built-in data logger within the UTM to maintain the load and displacement during the test. Total displacement for slippage and steel elongation were recorded. The braking force or the maximum load exerted to remove the steel rebar from the concrete was provided by the machine. The force was applied until the yielding of the steel bar or the bar slipped out from the specimen. Figure 6 shows the test setup, direction of loading, and specimen geometry. The ultimate bond stress of the concrete was calculated using Equation (1).

(1) $$\tau_{u}=\frac{P_{max}}{\pi \;L_{d}\;d_{b}}$$

where $\tau_{u}$ = ultimate bond stress of the concrete, $P_{max}$ = the peak force in the bar, $d_{b}$= diameter of rebar used, $L_{d}$ = depth of rebar penetration. The samples used in the pullout experiments were coded. For example, 0.00%-10-9$d_{b}$, where the first three numbers refer to the percentage content of GnP (0.00%), (10) refers to the bar diameter, and (9$d_{b}$), refers to the embedded length.

## 3. Experimental Results

### 3.1. The Compressive Strength of HPC-GnP Mixes

**Figure 7:**
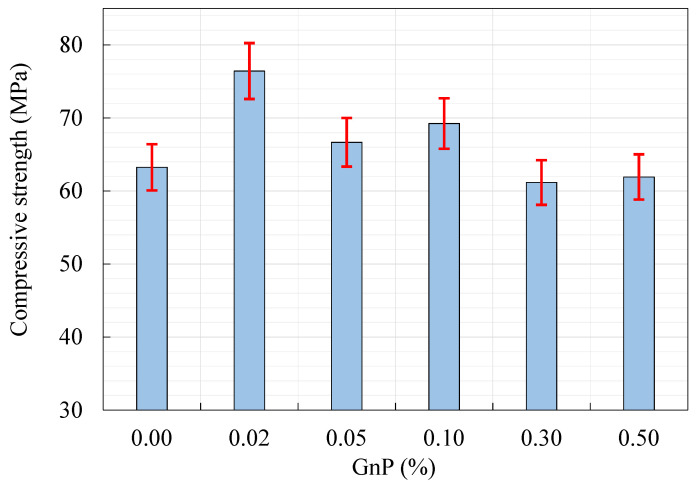
Compressive strength of HPC-GnP mixes.

The compressive strength of HPC samples with the mixtures listed in Table 1 is reported in Table 6. As it can be observed, the GnP content has a substantial impact on the compressive strength of HPC samples, with compressive strength increasing at first and subsequently decreasing as GnP content increased. As a result, there is a percentage of weight at which the optimum mechanical properties may be attained. In the current mix design, GnP with 0.02% content had the most effective positive impact on the compressive strength of the HPC. Figure 7 depicts the change in compressive strength of the HPC-GnP mixes. When compared to the base specimen (HPC0.00), it can be shown that adding 0.02, 0.05, and 0.10% of GnP to the HPC enhances its compressive strength by 20.82%, 5.41%, and 9.49%, respectively. These amounts of GnP reduced the HPC’s porosity, resulting in a more compact and continuous HPC nanocomposite. In this context, including GnP into the concrete matrix improves the connection between GnP and the concrete matrix around it, allowing GnP to be considered as the primary load-bearing component by providing more contact surfaces and therefore more force transmission surfaces. Moreover, it can be observed that adding 0.30 and 0.50% GnP to the HPC insignificantly decreases the compressive strength of the HPC (i.e., by 3.29% and 2.1%, respectively). As the GnP dose increased, the shear stress of the fresh HPC-GnP rose, resulted in low workability.

### 3.2. Bar Pullout Test 

Table 7 displays the results of the bar pullout test and identifies the failure modes for each specimen. Table 7 provides information on peak measured forces and related nominal bond stresses. The attained pullout force is divided by the starting surface area of the immersed section of the bar to determine the peak nominal bond stress, as shown in Equation (1). Failures due to rebar fracture, pullout, or splitting of the concrete sample should be noted. In the case of specimens that failed due to steel yielding, rather than reinforcement bar pullout, the ultimate bond stress estimated corresponds to the load at which yield occurs, which is less than the actual ultimate bond stress.

#### 3.2.1. Pullout Failure Behavior of Specimens

**Figure 8:**
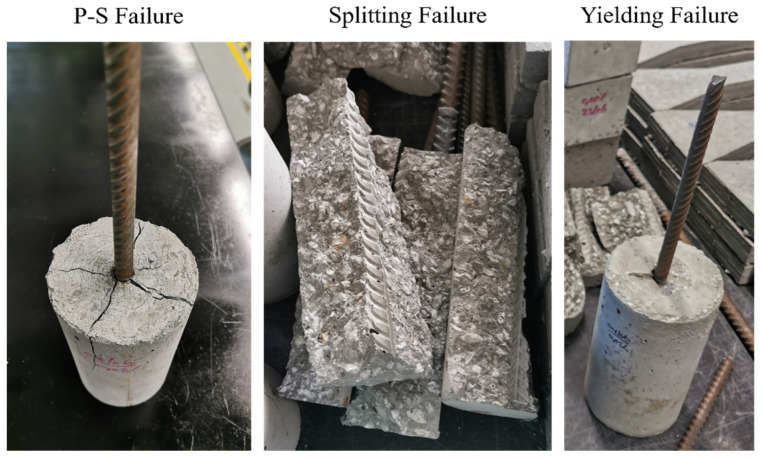
Pullout test samples with various failure mechanisms.

As seen in Table 7, there were three possible causes of failure for all of the samples. These modes can be addressed as follows: (1) combined failure mechanism of pulling out and splitting conical cracks [denoted (P-S)]; (2) splitting mode failure; (3) yielding failure. Figure 8 shows images of samples that failed the pullout test in any of the three mechanisms described above. The first mode failed as a result of reduced bearing capacity between the concrete and subsequent deformations of the bar. It was accompanied by radial cracks and crushing of the concrete near the bar. Those cracks extended to the upper longitudinal portion of the sample. The second type of failure occured mostly in the specimens which had a 16-mm diameter steel bar. In the HPC-GnP composite sample, the longitudinal cracks spread vertically; that is, they propagated vertically, without having a concrete cover, and the embedding length and capacity were increased. Finally, because of high bonding, bars of several specimens were yielded. A similar yielding failure is seen in the same figure, in which the steel bar achieved its maximum stress before reaching the ultimate bond stress. In the instance of yielding failure, there were only minor surface cracks immediately beyond the reinforcing bar. Table 7 shows that the samples without GnP failed with a (P-S) failure mode, whereas the samples with GnP failed with a steel yielding manner. For this observation, GnP enhanced the cohesiveness between the steel bar and concrete, resulting in a pullout force that was greater than the steel failure load. Moreover, the enhancement of GnP inclusion can be observed through the changing of the failure mode of the 16 mm bar diameter from (P-S) to a splitting mode.

#### 3.2.2. Bond Stress Slip Behavior 

**Figure 9:**
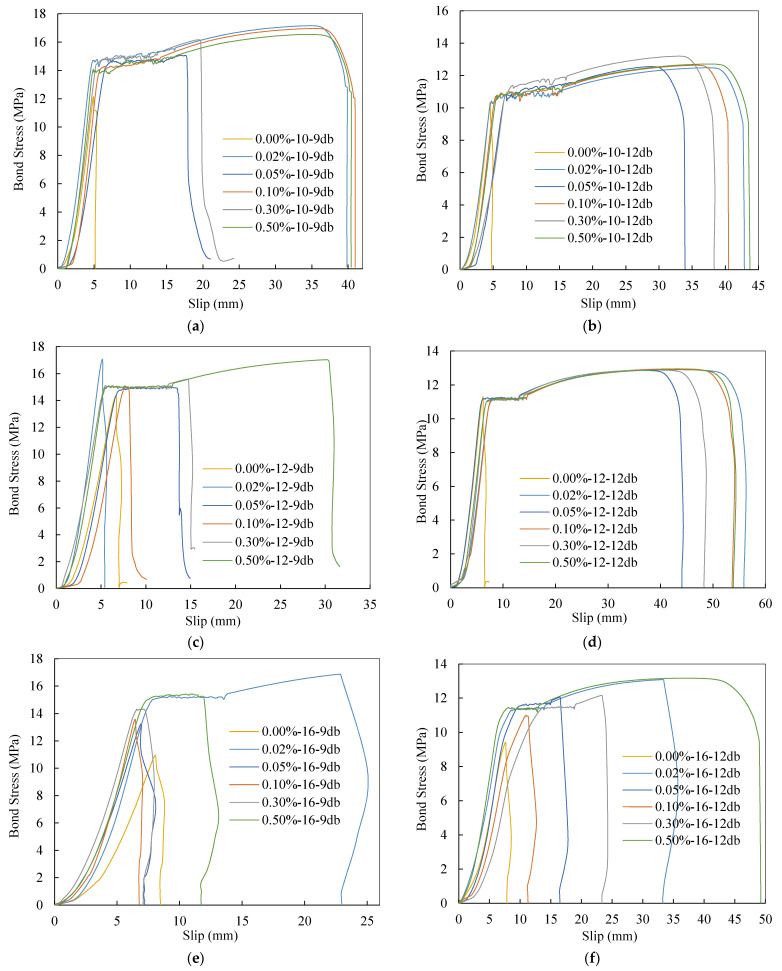
Slip behavior of specimens versus bond stress: (**a**) $d_{b}=$ 10 mm with $L_{d}=$ 90 mm; (**b**) $d_{b}=$ 10 mm with $L_{d}=$ 120 mm; (**c**) $d_{b}=$ 12 mm with $L_{d}=$ 108 mm; (**d**) $d_{b}=$ 12 mm with $L_{d}=$ 144 mm; (**e**) $d_{b}=$ 16mm with $L_{d}=$ 144 mm; (**f**) $d_{b}=$ 16mm with $L_{d}=$ 192 mm.

Figure 9 shows the slip–bond stress relationship for specimens. Slip is the displacement of the reinforcing bar relative to the surrounding concrete. According to Figure 9, all specimens initially displayed linear load-slip behavior before developing microcracks. Rebar begins to slip after microcracks are developed, and the bond stress–slip curve becomes less rigid. With the inclusion of GnP, it was discovered that some of the samples that failed in the pullout or splitting modes had less initial slippage due to the presence of GnP. Furthermore, some of the pullout and splitting failures, for example, 0.05%-10-9$d_{b}$, 0.02%-16-12$d_{b}$, were marked by continuous growth in bond stress–slip behavior up to the ultimate load, indicating a stable failure pattern. While the bond stress–slip behavior could not be attained beyond peak load in yielding failure because the bars broke before reaching the ultimate bond stress. The maximum slip was lower in specimens with smaller diameter bars than in specimens with 16 mm bars that failed due to bar pullout and splitting. Furthermore, because of the reduced concrete cover, longer embedding length, and increased bond stress, HPC-GnP for the 16 mm steel bar failed in a brittle manner. The specimens broke abruptly, creating longitudinal splitting fractures as shown in Figure 8. Based on the mechanisms of fractures, this explanation makes sense. Microcracking propagates in the material at a relative strain of 70–80% of the ultimate bonding stress due to the energy requirements of the fracture process. When a first fracture occurs at the steel-concrete contact, it quickly spreads as energy accumulates. Longitudinal splitting fractures occur fast, causing the connection to shatter in a brittle and abrupt manner [46].

#### 3.2.3. Effect of GnP on the Bond Stress

**Figure 10:**
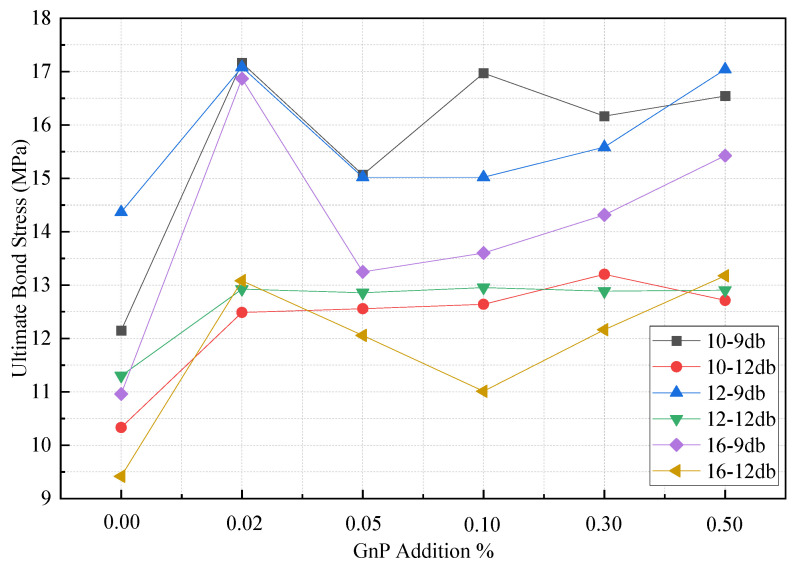
Effect of GnP inclusion on the ultimate bond stress.

GnP was employed to enhance the mechanical properties and filling characteristics of HPC in the current investigation. The effect of modulating the characteristics of HPC with GnP on bar-to-concrete adhesion has not been investigated. The findings of the pullout test for HPC-GnP are described in this section. According to the results indicated in Table 7, the inclusion of GnP in the HPC mix increased the ultimate bond stress by 24.02% to more than 41.28% for 0.05 wt.% and 0.02 wt.%, respectively, for a 10-mm steel bar diameter. Moreover, for a 12-mm steel bar diameter, the inclusion of GnP enhanced the ultimate bond stress by 18.91% for 0.02 wt.% compared to the reference sample. For 16-mm steel bar diameter, GnP increased the ultimate bond stress by 16.91% to 53.90% for 0.10 wt.% and 0.02 wt.%, respectively. Furthermore, the bond stress was significantly increased for 0.05, 0.10, 0.30, and 0.50 wt.% of GnP. However, 0.02 wt.% demonstrated the best performance in terms of increasing bond stress, rather than greater percentages. It is the suggested content from a cost perspective. Additionally, Table 6 shows that 0.30 and 0.50 wt.% of GnP reduced the compressive strength, while Table 7 shows that 0.30 and 0.50 wt.% of GnP increased the ultimate bond stress, which could be explained by the fact that the inclusion of GnP at higher doses enhanced adhesion between the steel bar and adjacent concrete. As shown in Figure 10, the HPC treated with GnP had considerably improved bond behavior when using GnP. The higher adhesion between the concrete and steel bar can be attributed to the improved bond behavior of the rebars and HPC owing to the inclusion of GnP.

#### 3.2.4. Bar Diameter Effect

**Figure 11:**
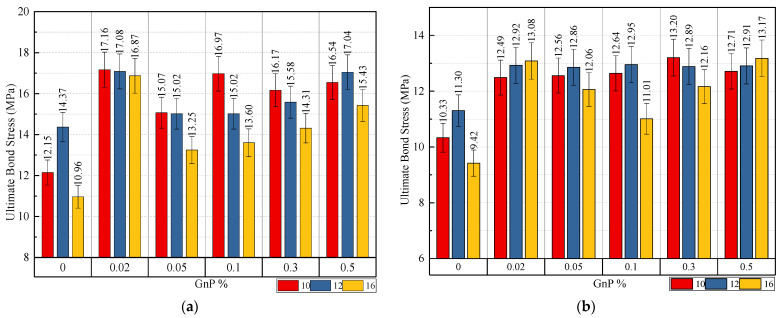
Impact of the diameter of the steel bar on ultimate bond stress for specimens with embedded length: (**a**) 9$d_{b}$; (**b**) 12$d_{b}$.

Figure 9 and Figure 11 demonstrate how bar diameter affects ultimate slip and bond stress, respectively. It is demonstrated that as the bar diameter grows, the bond stress decreases while the slippage increases. For specimens with embedded lengths of 9$d_{b}$, the ultimate bond stress for 0.10 wt.% of GnP is reduced by 11.49% and 9.45% when the bar diameter is increased from 10 to 12 and 16 mm, respectively. With a bar diameter of 10 mm, it is possible to obtain steel yielding properties with an embedded length of 12 mm, however the bar diameter was not detected since samples with 10 mm and 12 mm failed. Moreover, it shows that the contribution of GnP to bond stress was significantly greater for larger diameter bars (16 mm) than for smaller diameter bars (10 mm, 12 mm). It is thus fair to say that when GnP is added to bars of bigger diameter, bond stress rises as a result. The GnP combination action, which is responsible for both micro- and macro-level regulation of fracture development and propagation, plays an important role in this.

#### 3.2.5. Embedment Length Effect

**Figure 12:**
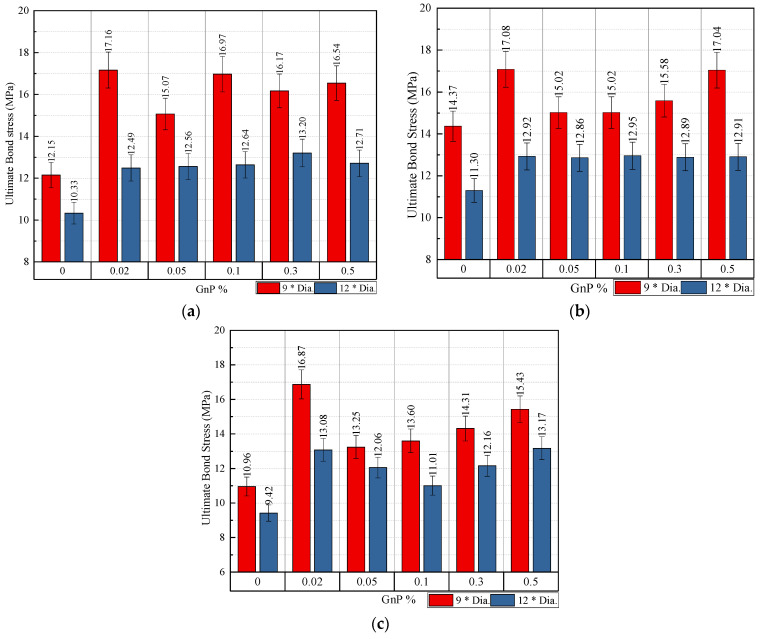
Impact of embedded length on ultimate bond stress for specimens with bar diameter: (**a**) 10 mm; (**b**) 12 mm; (**c**) 16 mm.

Figure 9 and Figure 12 demonstrate the influence of embedded length on ultimate slip and ultimate bond stress, respectively. As seen in the experiment, as the embedded length rises, the final bond tension decreases, and the corresponding slip increases. For example, when the embedded length increases from 9$d_{b}$ to 12$d_{b}$, the bond stress decreases by 22.45% for samples with 0.02%-16-9$d_{b}$, 0.02%-16-12$d_{b}$, and the slippage increases by 12.28%. For the control samples with a diameter of 10 and 12 mm, the stress was reduced by 15.05% and 21.36%, respectively. An example is shown in Figure 11a, which illustrates the effects of embedded length on the basic bar pullout for a 10-mm diameter bar with embedment lengths of 9$d_{b}$ and 12$d_{b}$. At 9$d_{b}$ most specimens failed through bar fracture with the inclusion of GnP and at 12$d_{b}$ all specimens failed with bar fracture, which means, the safely embedded length, in this case, falls between 9$d_{b}$ and 12$d_{b}$. Figure 11b, meanwhile, illustrates the effect of embedding length on the pullout of a 12-mm diameter with 9$d_{b}$ & 12$d_{b}$ embedments. At 9$d_{b}$ all specimens failed through pullout failure with the inclusion of GnP, and at 12$d_{b}$ all specimens failed with bar fracture, which means, the safely embedded length, in this case, falls between 9$d_{b}$ & 12$d_{b}$. In addition to that, in Figure 12c for the steel bar 16-mm diameter at 9$d_{b}$ & 12$d_{b}$, most of the samples failed in pullout failure. However, it can be seen that through the pullout failure load of 12$d_{b}$ with 16-mm diameter in Table 7, the failure load of the specimen (126.25 KN) for (GnP 0.02 wt.%) was near to the yield load (127.15) for (GnP 0.50 wt.%) of the steel bar, which means the safely embedded length is approximately 12$d_{b}$.

## 4. Comparison between Prediction Models and Experimental Results

The bonding between the reinforcing bars and the concrete has been studied by several researchers. A selection of these models is described below. Using the following formula, Orangun et al. [3] proposed:

(2) $$\tau_{u}=\left(0.10+0.25\frac{c_{min}}{d_{b}}+4.15\frac{d_{b}}{L_{d}}\right)\sqrt{f_{c}^{\prime }}$$

where $c_{min}$ is the minimum concrete cover in mm, $d_{b}$ is the diameter of the steel reinforcement bars in mm, $L_{d}$ is the embedded length of the bar, and $f_{c}^{\prime }$ is the compressive strength of concrete for cylinder sample. The ACI committee 408R [2] proposed the following formula:

(3) $$\tau_{u}=\left(\;1.43L_{d}\left(c_{min}+0.5d_{b}\right)+57.4A_{b}\right)\left(\frac{0.10c_{max}}{c_{min}}+0.90\right)\left(\frac{f_{c}^{\prime }{}^{0.25}}{\pi d_{b}L_{d}}\right)$$

where $c_{max}$ is the minimum concrete cover in mm and $A_{b}$ is the area of steel bar in $mm^{2}$. To evaluate the bond stress of high-strength concrete, Hadi [13] suggested the following formula for pullout testing:

(4) $$\tau_{u}=0.083045\sqrt{f_{c}^{\prime }}\;\left(22.8-0.208\left(\frac{c}{d_{b}}\right)-38.212\left(\frac{d_{b}}{L_{d}}\right)\right)$$

where c is the minimum concrete cover in mm. The following formula for calculating the bond stress was suggested by Esfahani and Rangan [6] for HPC having a compressive strength of 50 MPa or above:

(5) $$\tau_{u}=8.6\;f_{ct}\left(\frac{(c/d_{b})+0.5}{(c/d_{b})+5.5}\right)\;$$

where $f_{ct}$ is the tensile strength of concrete and taken as $0.55\sqrt{f_{c}^{\prime }}$. Another formula for calculating the bond stress was suggested by Chapman and Shah [14]:

(6) $$\tau_{u}=\left(0.29+0.282\frac{c_{min}}{d_{b}}+4.734\frac{d_{b}}{L_{d}}\right)\sqrt{f_{c}^{\prime }}$$

**Figure 13:**
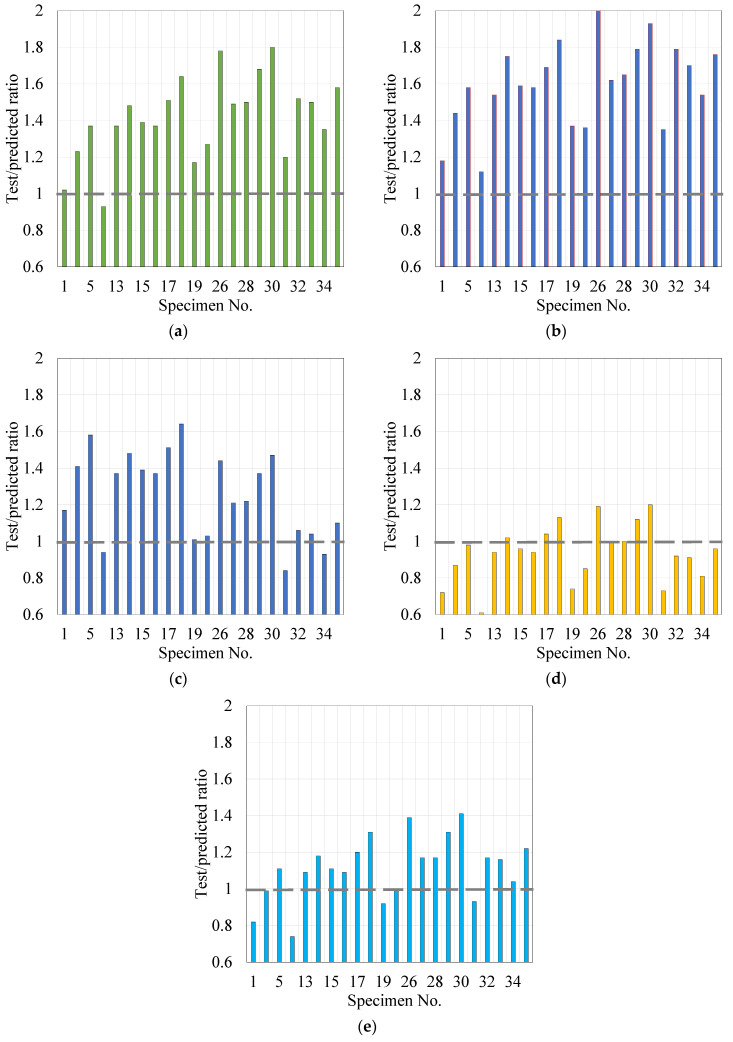
Bond stress test results/prediction ratios versus the specimen number (Table 7): (**a**) Orangun et al. [3]; (**b**) ACI committee 408R [2]; (**c**) Hadi [13]; (**d**) Esfahani and Rangan [6]; (**e**) Chapman and Shah [14].

Table 8 shows the obtained bond stress results (Equation (1)) and the predicted bond stress using the equations of Orangun et al. [3], ACI committee 408R [2], Hadi [13], Esfahani and Rangan [6], and Chapman and Shah [14] (Equations (2)–(6)). The comparison ratios between experimental and predicted results are illustrated in Table 9 and Figure 13. The proposed equations by Esfahani and Rangan, and Chapman and Shah match the test findings more closely than the other prediction equations. The mean ratio of experimental results to the equation of Esfahani and Rangan, and Chapman and Shah are 0.94, 1.12 with a standard deviation of 0.15 and 0.17, respectively. Accordingly, the ultimate bond stress values are higher than those anticipated by the Orangun et al., ACI, and Hadi equations, where the mean ratios of experimental results to the Orangun et al., ACI, and Hadi equations are 1.42, 1.60, and 1.25 with standard deviations of 0.22, 0.22, and 0.23, respectively. As a result, the preceding calculations of Orangun et al., ACI, and Hadi underestimated the bond stress. However, the prediction equation of Esfahani and Rangan overestimated the bond stress of control samples, but it was able to predict the bond stress of those samples, which had the GnP incorporation, with a mean ratio of 0.99 and a standard deviation of 0.12.

## 5. Conclusions

The bond stress behavior of HPC containing GnP was investigated in the current study using 36 samples. The bond stress and slip behavior between the rebar and concrete were evaluated and discussed after a pullout test on numerous experimental specimens. Furthermore, the influence of variables such as the diameter of the bar, embedded length of the bar, and percentage of GnP on the bond stress was assessed. Based on the findings of this research, the following conclusions may be drawn:

In comparison to HPC without GnP, the results indicated that HPC with GnP had improved bond stress due to the bridging and confinement action of GnP as a nano reinforcement.

The results showed that the inclusion of 0.02 wt.% of GnP enhanced the bond stress by more than 41.28% for steel bars with 10 mm and 18.91% and 53.90% for steel bars with 12 and16 mm, respectively, at the same embedded length 9$d_{b}$.

In comparison to the control samples, the inclusion of GnP caused a reduction in the initial slippage of the steel bar due to the enhanced adhesion between the bar and adjacent concrete.

However, the excessive dosage of GnP caused a reduction in the compressive strength of HPC, GnP at high doses (0.50 wt.%) showed improved bond stress, which was near to the same enhancement of 0.02 wt.% of GnP.

The use of GnP reinforced HPC can lead to a decrease in the length of anchoring required for deformed bars.

Esfahani and Rangan’s prediction equation was able to nearly predict the bond stress of GnP-incorporated samples with a mean ratio of 0.99 and a standard deviation of 0.12.

## Figures and Tables

**Table 1:** Details of concrete designations and variables.

No	Designation	GnP	$d_{b}\;(\mathrm{mm})$	$L_{d}\;(\mathrm{mm})$
1	HPC0.00	0.00%	10, 12, 16	$(9d_{b}\;\&\;12d_{b}$)
2	HPC0.02	0.02%	10, 12, 16	$(9d_{b}\;\&\;12d_{b}$)
3	HPC0.05	0.05%	10, 12, 16	$(9d_{b}\;\&\;12d_{b}$)
4	HPC0.10	0.10%	10, 12, 16	$(9d_{b}\;\&\;12d_{b}$)
5	HPC0.30	0.30%	10, 12, 16	$(9d_{b}\;\&\;12d_{b}$)
6	HPC0.50	0.50%	10, 12, 16	$(9d_{b}\;\&\;12d_{b}$)

**Table 2:** Properties of Cement.

Properties		Test Result
CaO		81.20%
SiO_2_		8.59%
Fe_2_O_3_		3.18%
SO_3_		2.78%
Al_2_O_3_		2%
K_2_O		0.72%
MgO		0.68%
Initial time setting		155 min
Compressive strength	7 d	24 MPa
	28 d	≤52.5 MPa

**Table 3:** Properties of Silica Fume.

Properties	Test Result
CaO	1.51%
SiO_2_	94.24%
Fe_2_O_3_	1.33%
Al_2_O_3_	0.57%
MgO	0.42%
Density (g/cm^3^)	2.28
Fineness (m^2^/kg)	22,000
LOI	0.84%

**Table 4:** Properties of GnP.

Product	Density (g/cm^3^)	Diameter (µm)	Thickness (nm)	Surface Area (m^2^/g)	Carbon Content (%)	Elastic Modulus (GPa)	Tensile Strength (GPa)
xGnP-C300	0.20–0.40	≈2	≈2	300	99.52	1000	5

**Table 5:** HPC M60 mix proportion.

Water kg/m³	Cement kg/m³	Silica Fume kg/m³	Coarse Aggregate kg/m³	Fine Aggregate kg/m³	Plasticizer L/m³
165.8	500	35	1072.3	660.2	10

**Table 6:** Compressive strength of HPC with different content of GnP.

No	Designation	% GnP	Cube Compressive Strength (MPa)	% Difference
1	HPC0.00	0.00%	63.25	-
2	HPC0.02	0.02%	76.42	+20.82
3	HPC0.05	0.05%	66.67	+5.41
4	HPC0.10	0.10%	69.25	+9.49
5	HPC0.30	0.30%	61.17	−3.29
6	HPC0.50	0.50%	61.92	−2.10

**Table 7:** Test Results for Simple Bar Pullout.

No.	Designation	GnP	$d_{b}\;(\mathrm{mm})$	$L_{d}\;(\mathrm{mm})$	$P_{max}\;(\mathrm{kN})$	$\tau_{u}\;(\mathrm{MPa})$	$\%\;\mathrm{of}\;\tau_{u}\;\mathrm{Increase}$	Mode of Failure
1	$0.00\%-10-9d_{b}$	0.00%	10	90	34.35	12.15	-	P-S
2	$0.02\%-10-9d_{b}$	0.02%	10	90	48.53	17.16 *	>41.28	Steel Yield
3	$0.05\%-10-9d_{b}$	0.05%	10	90	42.60	15.07	24.02	P-S
4	$0.10\%-10-9d_{b}$	0.10%	10	90	47.98	16.97 *	>39.68	Steel Yield
5	$0.30\%-10-9d_{b}$	0.30%	10	90	45.71	16.17	33.07	P-S
6	$0.50\%-10-9d_{b}$	0.50%	10	90	46.77	16.54 *	>36.16	Steel Yield
7	$0.00\%-10-12d_{b}$	0.00%	10	120	38.95	10.33	-	P-S
8	$0.02\%-10-12d_{b}$	0.02%	10	120	47.08	12.49 *	>20.87	Steel Yield
9	$0.05\%-10-12d_{b}$	0.05%	10	120	47.34	12.56 *	>21.54	Steel Yield
10	$0.10\%-10-12d_{b}$	0.10%	10	120	47.65	12.64 *	>22.34	Steel Yield
11	$0.30\%-10-12d_{b}$	0.30%	10	120	49.77	13.20 *	>27.78	Steel Yield
12	$0.50\%-10-12d_{b}$	0.50%	10	120	47.93	12.71 *	>23.06	Steel Yield
13	$0.00\%-12-9d_{b}$	0.00%	12	108	58.49	14.37	-	P-S
14	$0.02\%-12-9d_{b}$	0.02%	12	108	69.55	17.08	18.91	P-S
15	$0.05\%-12-9d_{b}$	0.05%	12	108	61.15	15.02	4.55	P-S
16	$0.10\%-12-9d_{b}$	0.10%	12	108	61.15	15.02	4.55	P-S
17	$0.30\%-12-9d_{b}$	0.30%	12	108	63.45	15.58	8.48	P-S
18	$0.50\%-12-9d_{b}$	0.50%	12	108	69.38	17.04	18.62	P-S
19	$0.00\%-12-12d_{b}$	0.00%	12	144	61.35	11.30	-	P-S
20	$0.02\%-12-12d_{b}$	0.02%	12	144	70.16	12.92 *	>14.36	Steel Yield
21	$0.05\%-12-12d_{b}$	0.05%	12	144	69.80	12.86 *	>13.77	Steel Yield
22	$0.10\%-12-12d_{b}$	0.10%	12	144	70.32	12.95 *	>14.62	Steel Yield
23	$0.30\%-12-12d_{b}$	0.30%	12	144	69.95	12.89 *	>14.02	Steel Yield
24	$0.50\%-12-12d_{b}$	0.50%	12	144	70.06	12.91 *	>14.20	Steel Yield
25	$0.00\%-16-9d_{b}$	0.00%	16	144	79.33	10.96	-	P-S
26	$0.02\%-16-9d_{b}$	0.02%	16	144	122.09	16.87	53.90	Splitting
27	$0.05\%-16-9d_{b}$	0.05%	16	144	95.88	13.25	20.86	Splitting
28	$0.10\%-16-9d_{b}$	0.10%	16	144	98.44	13.60	24.09	Splitting
29	$0.30\%-16-9d_{b}$	0.30%	16	144	103.61	14.31	30.61	Splitting
30	$0.50\%-16-9d_{b}$	0.50%	16	144	111.65	15.43	40.74	Splitting
31	$0.00\%-16-12d_{b}$	0.00%	16	192	90.90	9.42	-	P-S
32	$0.02\%-16-12d_{b}$	0.02%	16	192	126.25	13.08	38.89	Splitting
33	$0.05\%-16-12d_{b}$	0.05%	16	192	116.36	12.06	28.01	Splitting
34	$0.10\%-16-12d_{b}$	0.10%	16	192	106.27	11.01	16.91	Splitting
35	$0.30\%-16-12d_{b}$	0.30%	16	192	117.39	12.16	29.14	Splitting
36	$0.50\%-16-12d_{b}$	0.50%	16	192	127.15	13.17 *	>39.87	Steel Yield

$P_{\max}$: Pullout Load; $\tau_{u}$: Ultimate Bond Stress; P-S: Combined failure mechanism of pulling out and splitting conical cracks; Splitting: splitting mode failure. * The ultimate load at yielding failure is smaller than the actual bond stress.

**Table 8:** The estimated bond stress using prediction equations compared to the bond stress obtained experimentally.

	Dimensions (mm)			(MPa)	Ultimate Bond Stress (MPa)					
Sample Des.	$d_{b}$	$L_{d}$	*c*	$f_{c}^{\prime }$	$\tau_{u}\;(1)\;\mathrm{Exper}$	Orangun (2)	ACI 408R (3)	Hadi (4)	Esfahani, Rangan (5)	Chapman, Shah (6)
$0.00\%-10-9d_{b}$	10	90	45	50.60	12.15	11.97	10.32	10.41	16.82	14.83
$0.02\%-10-9d_{b}$	10	90	45	61.14	17.16	13.15	10.82	11.44	18.49	16.30
$0.05\%-10-9d_{b}$	10	90	45	53.34	15.07	12.28	10.46	10.69	17.27	15.23
$0.10\%-10-9d_{b}$	10	90	45	55.40	16.97	12.52	10.56	10.89	17.60	15.52
$0.30\%-10-9d_{b}$	10	90	45	48.94	16.17	11.77	10.23	10.24	16.54	14.59
$0.50\%-10-9d_{b}$	10	90	45	49.54	16.54	11.84	10.27	10.30	16.65	14.67
$0.00\%-10-12d_{b}$	10	120	45	50.60	10.33	11.15	9.26	11.03	16.82	13.90
$0.02\%-10-12d_{b}$	10	120	45	61.14	12.49	12.25	9.71	12.13	18.49	15.27
$0.05\%-10-12d_{b}$	10	120	45	53.34	12.56	11.44	9.38	11.33	17.27	14.27
$0.10\%-10-12d_{b}$	10	120	45	55.40	12.64	11.66	9.47	11.55	17.60	14.54
$0.30\%-10-12d_{b}$	10	120	45	48.94	13.20	10.96	9.18	10.85	16.54	13.67
$0.50\%-10-12d_{b}$	10	120	45	49.54	12.71	11.03	9.21	10.92	16.65	13.75
$0.00\%-12-9d_{b}$	12	108	44	50.60	14.37	10.49	9.31	10.51	15.29	13.16
$0.02\%-12-9d_{b}$	12	108	44	61.14	17.08	11.53	9.76	11.55	16.81	14.47
$0.05\%-12-9d_{b}$	12	108	44	53.34	15.02	10.77	9.43	10.79	15.70	13.51
$0.10\%-12-9d_{b}$	12	108	44	55.40	15.02	10.97	9.52	11.00	16.00	13.77
$0.30\%-12-9d_{b}$	12	108	44	48.94	15.58	10.31	9.23	10.34	15.04	12.94
$0.50\%-12-9d_{b}$	12	108	44	49.54	17.04	10.38	9.26	10.40	15.13	13.02
$0.00\%-12-12d_{b}$	12	144	44	50.60	11.30	9.67	8.25	11.14	15.29	12.22
$0.02\%-12-12d_{b}$	12	144	44	61.14	12.92	10.63	8.65	12.24	16.81	13.44
$0.05\%-12-12d_{b}$	12	144	44	53.34	12.86	9.93	8.36	11.43	15.70	12.55
$0.10\%-12-12d_{b}$	12	144	44	55.40	12.95	10.12	8.44	11.65	16.00	12.79
$0.30\%-12-12d_{b}$	12	144	44	48.94	12.89	9.51	8.18	10.95	15.04	12.02
$0.50\%-12-12d_{b}$	12	144	44	49.54	12.91	9.57	8.20	11.02	15.13	12.10
$0.00\%-16-9d_{b}$	16	144	42	50.60	10.96	8.64	8.05	10.64	12.94	11.07
$0.02\%-16-9d_{b}$	16	144	42	61.14	16.87	9.50	8.44	11.69	14.22	12.17
$0.05\%-16-9d_{b}$	16	144	42	53.34	13.25	8.87	8.15	10.92	13.29	11.37
$0.10\%-16-9d_{b}$	16	144	42	55.40	13.60	9.04	8.23	11.13	13.54	11.58
$0.30\%-16-9d_{b}$	16	144	42	48.94	14.31	8.50	7.98	10.46	12.73	10.89
$0.50\%-16-9d_{b}$	16	144	42	49.54	15.43	8.55	8.00	10.53	12.80	10.95
$0.00\%-16-12d_{b}$	16	192	42	50.60	9.42	7.82	6.98	11.27	12.94	10.13
$0.02\%-16-12d_{b}$	16	192	42	61.14	13.08	8.60	7.32	12.38	14.22	11.14
$0.05\%-16-12d_{b}$	16	192	42	53.34	12.06	8.03	7.08	11.57	13.29	10.41
$0.10\%-16-12d_{b}$	16	192	42	55.40	11.01	8.18	7.14	11.79	13.54	10.60
$0.30\%-16-12d_{b}$	16	192	42	48.94	12.16	7.69	6.93	11.08	12.73	9.97
$0.50\%-16-12d_{b}$	16	192	42	49.54	13.17	7.74	6.95	11.15	12.80	10.03

**Table 9:** Ratios for experimental test result/prediction of bond stress.

Sample Des.	(1)/(2)	(1)/(3)	(1)/(4)	(1)/(5)	(1)/(6)
$0.00\%-10-9d_{b}$	1.02	1.18	1.17	0.72	0.82
$0.05\%-10-9d_{b}$	1.23	1.44	1.41	0.87	0.99
$0.30\%-10-9d_{b}$	1.37	1.58	1.58	0.98	1.11
$0.00\%-10-12d_{b}$	0.93	1.12	0.94	0.61	0.74
$0.00\%-12-9d_{b}$	1.37	1.54	1.37	0.94	1.09
$0.02\%-12-9d_{b}$	1.48	1.75	1.48	1.02	1.18
$0.05\%-12-9d_{b}$	1.39	1.59	1.39	0.96	1.11
$0.10\%-12-9d_{b}$	1.37	1.58	1.37	0.94	1.09
$0.30\%-12-9d_{b}$	1.51	1.69	1.51	1.04	1.20
$0.50\%-12-9d_{b}$	1.64	1.84	1.64	1.13	1.31
$0.00\%-12-12d_{b}$	1.17	1.37	1.01	0.74	0.92
$0.00\%-16-9d_{b}$	1.27	1.36	1.03	0.85	0.99
$0.02\%-16-9d_{b}$	1.78	2.00	1.44	1.19	1.39
$0.05\%-16-9d_{b}$	1.49	1.62	1.21	1.00	1.17
$0.10\%-16-9d_{b}$	1.50	1.65	1.22	1.00	1.17
$0.30\%-16-9d_{b}$	1.68	1.79	1.37	1.12	1.31
$0.50\%-16-9d_{b}$	1.80	1.93	1.47	1.20	1.41
$0.00\%-16-12d_{b}$	1.20	1.35	0.84	0.73	0.93
$0.02\%-16-12d_{b}$	1.52	1.79	1.06	0.92	1.17
$0.05\%-16-12d_{b}$	1.50	1.70	1.04	0.91	1.16
$0.10\%-16-12d_{b}$	1.35	1.54	0.93	0.81	1.04
$0.30\%-16-12d_{b}$	1.58	1.76	1.10	0.96	1.22
Mean values *	1.42	1.60	1.25	0.94	1.12
Standard deviation	0.22	0.22	0.23	0.15	0.17

* Mean values and standard deviations were calculated based on the specimens which failed in the pullout mode, excluding those that failed due to steel yielding.

## Data Availability

All research data is available and can be furnished upon request.
